# 

**Published:** 1865-01

**Authors:** 


### INDEX TO VOLUME XXII.

Ascites and its Treatment—Baker. 547
American Medical Association.... 18
232, 325, 288
Address to the A. Dental Association—Prof. Brainard........... 356
American Dental Association..... 375
Arsenic-Eating.................. 408
Address, Valedictory—Prof. Carr.. 193
" Introductory–Prof. Blaney. 481
Adhesion, Extraordinary Case of.. 520
Bromide of Potassium............ 37
Books Received.............. 480, 528
Bone, Reproduction of........... 39
Brainard's (Prof.) Visit to Europe.. 565
Clinical Lectures on Diseases of the Eye—Holmes..... 11, 160, 206, 246
390, 441, 494, 551
Climate, Tropical effects of....... 77
Circular to the Medical Profession—Prof. Allen.................. 164
Chloroform................. 165, 374
" Deaths from.......... 168
Cancer, Surgical Treatment of.... 263
Cerebro-Spinal Meningitis—Ingals. 289
Cerebro-Spinal Meningitis—White. 529
Cerebro-Spinal Meningitis–Travers. 451
Chicago Republican on Obscene Advertisements.................. 323
Cork and its Uses................ 351
Croup, Treatment of............. 461
Circulation in Trees............. 462
Carbuncle....................... 469
Cholera, Deaths from............ 527
Diseases, Hereditary Transmission of 49
Diphtheria—Whitmire........... 140
Diarrhœa.............. 471, 241, 344
Dialysis........................ 251
Dysentery—Trimble.............. 337
Deformities, Treatment of—Blackall........................... 371
Diamonds, Origin of............. 410
Dysmenorrhea, &c., Treated by division......................... 411
Diabetes........................ 416
Disinfection.................... 459
Drunkenness, Cure for........... 470
Excision of Os Calcis and Astragalas—Baxter.................. 385
Empyemia—Whitmire............ 433
Erythema, Hereditary............ 522
Epilepsy........................ 318
Fracture of Femur—Baxter....... 33
Fogyism in Physic............... 57
Fluid Extracts.................. 458
Fever, Typhoid.................. 15
Fracture, Compound Comminuted, of Leg........................ 395
Glycerine, Uses of.............. 259
Gonorrhœa..................... 314
Graduates of Medical Colleges..... 384
Glaucoma...................... 432
Gun-Shot Wound................ 568
Gun-Shot Injuries of Head, Treatment of....................... 546
Hydrocephalus, Fatal............ 55
Hospital Gangrene.............. 68
" —McPherson........... 226
" Cook County.......... 564
Homœopathic Charges against Prof. Freer, Investigation of......... 98
Homeopathic Globules........... 210
Heart, Gun-Shot Wound of....... 299
" Fatty, from Smoking...... 310
Hints to Travelers............... 316
Hypodermic Administration of Remedies....................... 398
Hemiplegia..................... 492
Iodine in Neuralgia............. 91
Insane, Statistics of, Ohio......... 528
Inspiration of Vapor............ 44
Lead-poison.................... 32
Laudanum and Teetotalism....... 49

Lincoln, Last Hours of...........227
Longevity.......................317
Medical Society, Illinois ..10, 142, 275
Menstruation, Chinese Custom during.........................550
Milk Sickness...................139
MARRIAGE NOTICES.
J. L. Prentiss, M. D........238
F. C. Robinson, M. D.......384
Geo. W. Beggs, M. D.......384
Jos. H. Wilson, M. D......428
R. T. Richards, M. D........527
B. K. Shurtliff.............527
Medical Agents from the Body, Elimination of... ................571
Medical Society, DeWitt County..334
375
Fox River Valley Assoc'n.....376
Military Service, Causes of Exemption from....................567
Midwifery, Extremely Difficult Case of—Paoli.....................556
Morton Testimonial..............561
Nux Vomica in Asthenic Diseases..154
Nitric Acid in Syphilitic Cachexy..262
Nux Vomica in Diarrhœa.........479
Origin of Brandy................ 94
Orthopedic Surgery—Prince......122
Obituary—E. E. Lynn, M. D.......238
David Rutter, M. D....238
Joseph Lee, M. D......523
J. L. Riddle, M. D......572
Prof. J. F. Malgaigne...572
John Lindley, M. D.....572
Dr. Breard...........572
Wm. Lyman, M. D.....572
Puerperal Fever—Miller.......... 1
Projectiles...................... 41
Permonganate of Potash in Diphtheria.....................75, 312
Pathology of Deposits of Urates... 81
Physiology of Fasting...........172
Pneumonia, Treatment of.........304
Per Sulphate Iron in Hemorrhoids..316
Pluro Pneumonia.................399
Pathologia Generalis.............444
Price Current....................467
Physiology in Medicine and Surgery499
Preventive Medicine.............516
Puerperal Women, Position and Diet of—Hibberd.... .........533
Revaccination...................183
Relations of Therapeutics to Medicine.......................525
Rheumatism, Treatment of........470
" " ........479
Rush Medical College, Graduates of. 84
" " " Dispensary..427
" " " Demonstrator of.........................428
REVIEWS—
Handbook of Skin Diseases.....238
Braithwaite's Retrospect Index..239
Medical Lexicon—Roosa.......239
Practical Receipt Book, for Druggists—Branston........... .239
Proceedings of A. Phar. Ass'n...288
Dispensary, U. S..............288
Practical Surgery—Van Buren...382
Report of Provost Marshal Gen'l.383
Military Surgery—Hamilton.....429
A System of Surgery—Gross....430
Essentials in Materia Medica—Garrod....................431
Scabies.......................... 19
Speech, Mechanism of............174
Sugar and Lactic Acid...........211
Small Pox in the Fœtus..........235
Sickness in Europe..............254
Submarine Telegraph...........378
Syphilisation ...................454
Syncope, &c.....................465
Syphilis, Infantile...............469
Surgical Operation...............470
Southern Medical Journals........524
Tannin; its Use in Pruritus Vulva and Vagina...................559
Tapeworm Treated with Elm Bark.. 87
Tonsil, Exbition of..............153
Transformation of Muscular Fibre into Fat........................261
Tom Thumb......................384
Treasures Locked Up............523
Umbilical Hernia................570
Unusual Case, Report of—Meachem.157
Ulcers..........................526
Vaccine Virus, Preservation of—Prince.........................156
Vaccination and Re-Vaccination..176
Valentine Mott............237, 527
Weakness of Vision in the Aged..317

American Medical Association ........ 18

### REMOVAL.

☞ The Editorial and Private Office of DRS. MILLER and INGALS will hereafter be at No. 190 SOUTH CLARK ST., between Monroe and Adams Sts.

☞ Business letters to the JOURNAL should be addressed to Drs. MILLER & INGALS, Chicago, Ills., P. O. Drawer 5787. When money is received a receipt will be returned with the next number of the JOURNAL, and subscribers failing to get such receipt will confer a favor by giving us notice of any omission.

